# Framework for Intelligent Swimming Analytics with Wearable Sensors for Stroke Classification

**DOI:** 10.3390/s21155162

**Published:** 2021-07-30

**Authors:** Joana Costa, Catarina Silva, Miguel Santos, Telmo Fernandes, Sérgio Faria

**Affiliations:** 1 ESTG, Polytechnic of Leiria, 2411-901 Leiria, Portugal; 2172570@my.ipleiria.pt (M.S.); telmo.fernandes@ipleiria.pt (T.F.); 2 CISUC—Centre Informatics and Systems, Informatics Engineering Department, University of Coimbra, 3004-531 Coimbra, Portugal; catarina@dei.uc.pt (C.S.); sergio.faria@ipleiria.pt (S.F.); 3 Instituto de Telecomunicações, 2400-835 Leiria, Portugal

**Keywords:** wearable sensors, data acquisition, sensor data representation, feature representation, intelligent systems, ensemble methods

## Abstract

Intelligent approaches in sports using IoT devices to gather data, attempting to optimize athlete’s training and performance, are cutting edge research. Synergies between recent wearable hardware and wireless communication strategies, together with the advances in intelligent algorithms, which are able to perform online pattern recognition and classification with seamless results, are at the front line of high-performance sports coaching. In this work, an intelligent data analytics system for swimmer performance is proposed. The system includes (i) pre-processing of raw signals; (ii) feature representation of wearable sensors and biosensors; (iii) online recognition of the swimming style and turns; and (iv) post-analysis of the performance for coaching decision support, including stroke counting and average speed. The system is supported by wearable inertial (AHRS) and biosensors (heart rate and pulse oximetry) placed on a swimmer’s body. Radio-frequency links are employed to communicate with the heart rate sensor and the station in the vicinity of the swimming pool, where analytics is carried out. Experiments were carried out in a real training setup, including 10 athletes aged 15 to 17 years. This scenario resulted in a set of circa 8000 samples. The experimental results show that the proposed system for intelligent swimming analytics with wearable sensors effectively yields immediate feedback to coaches and swimmers based on real-time data analysis. The best result was achieved with a Random Forest classifier with a macro-averaged $F_{1}$ of 95.02%. The benefit of the proposed framework was demonstrated by effectively supporting coaches while monitoring the training of several swimmers.

## 1. Introduction

Ubiquitous sensing opportunities are kindling interest in many areas, where improving performance in sports is a paradigmatic example. Recently, different approaches for developing wearable sensors for sports are being sought [1,2,3,4]. However, initial methodologies were developed essentially for monitoring purposes, and initial approaches employing intelligent systems in sports analytics, namely in swimming, have neglected the possibility of using wearable sensors, focusing essentially on statistics or crowdsourced data [5] and recorded video [6,7,8,9].

On the other hand, the opportunity to integrate intelligent pattern recognition techniques and prediction ability brings an enormous potential to cope with more ambitious challenges, namely to achieve athlete performance improvements through intelligent analytics [10,11,12].

Nevertheless, designing and implementing a framework that effectively supports and promotes sports performance is a challenging task for two reasons. First, a real-time acquisition and communication architecture must be developed. Second, a robust and efficient intelligent system that can support coaches’ decisions in real time must be conceived.

Sensors, both wearable inertial sensors and biosensors, e.g., heart rate, can be efficient tools for intelligent swimming analytics [1]. However, despite the recent evolution of IoT, there is still a lack of off-the-shelf and easy-to-use performance monitoring systems, not only due to the constant hardware and communications technology evolution but also because these fail to build trust amongst coaches and athletes. Additionally, although research on wearable sensor development is present in the literature, as detailed below, few intelligent swimming analytics approaches can be found.

Swimming is a highly competitive sport, with world-class athletes aiming to optimize performance [1]. Within this context, coaches focus on constantly keeping track of swimmers, using all available methods to analyse their performance and to define enhancement strategies.

Cutting-edge intelligent systems often present shortcomings in terms of efficiency. Examples of such limitations include those resulting from excessive processing times, leading to output result delays. The presented approach aims to mitigate these problems using ensemble strategies to achieve two main goals: first, by combining different state-of-the-art classifiers, results can be achieved and even exceeded [13,14]; second, the essential parallelizable architecture of ensembles make them particularly adequate for real-time set up.

Moreover, the combination of computationally demanding intelligent methods with the need for real time assessments, eventually on mobile devices, also leads to the development of flexible solutions that adapt to dynamic scenarios in terms of computational needs and features to be made available, e.g., performance classification or forecast of upcoming results. Intelligent methods such as Naïve Bayes, K-Nearest Neighbors, Decision Trees, Random Forests, or Support Vector Machines may be combined in ensembles, effectively responding to these flexible and heterogeneous scenarios [14].

In this paper, a swimmer performance intelligent data analytics system is proposed, which includes (i) pre-processing of raw signals; (ii) feature representation of wearable inertial sensors and biosensors; (iii) online recognition of the swimming style and turns; and (iv) post-analysis of the performance for coaching decision support, including average speed and stroke counting.

The present paper is organized as follows. The next section introduces related work on the fundamentals sensors used in sports, supporting the proposed framework. Section 3 addresses the wearable sensors and the employed communication architecture. Then, the method is detailed in two sections: Section 4 focuses on the proposed framework for swimming analytics, including the feature representation of sensor data, and Section 5 describes the experimental setup. Section 6 and Section 7 present and discuss the experimental results, and finally, Section 8 concludes and draws future lines of research.

## 2. Sensors in Sports

Nowadays, sports—especially high-performance sports—are a thriving field for sensorization. In the following, there is a description of the current approaches and advancements in data acquisition technologies and performance monitoring systems that are set up and explored, with a particular focus on swimming.

### 2.1. Data Acquisition Technologies

When focusing on swimming, there are some efforts on research and development described in the literature using different data acquisition methods.

The most conventional approach used in the majority of the research works is based on inertial sensors placed on the athlete, including a Micro Controller Unit (MCU) inside a sealed waterproof case [4,15,16].

A less invasive approach is to employ video cameras, usually placed underwater in the swimming pool [17,18]. Such video systems can be installed in the field with no need for encumbering the athletes with carrying additional hardware and are able to track motion and to analyse the performance from a mechanical perspective.

Nevertheless, wearable devices are both less expensive and easier to set up in different scenarios and locations and allow for the acquisition of physiological data such as heart rate, breath rate, body temperature, motion, and position information, among others.

Additionally, nowadays, there is a wide diversity of wireless technologies that free the wearable devices from the need to be physically attached to a computer or a datalogger. Such advances drastically reduce the number of required wires in the system, contributing to the athletes’ freedom in terms of motion and therefore reducing the system impact in their performance during training.

### 2.2. Performance Monitoring Systems

Regarding performance monitoring systems, in [1], inertial measurement units (IMU) were used in a macro–micro analysis approach comprehensive enough to cover a full training session, regardless of the swimming technique used at any particular time. Several swimmers were monitored and the approach detected swimming bouts, laps, and swimming technique at the macro level. A statistic correlation between the sensor values and the output results was proposed.

In [3], a pervasive monitoring solution for physiological and biomechanical signals was proposed. A wearable IMU was used for data acquisition and later processing. The main contribution is related to the commercial availability of such system for real time analysis by coaches and teams.

Although sensor data were not used, in [5], statistical analysis and machine learning methods were systematically applied to swimming records, investigating performance features for all strokes as a function of age and gender. It included two novel features, namely the use of machine learning methods and the use of an ensemble classifier that outperforms the single instance intelligent classifiers.

Finally, it is worth mentioning that a survey was presented in [6], which included several swimming coaches and teams in the USA. The performance analysis practices of swimming coaches were examined, studying the reasons behind the coaches’ decisions. As a result, a general picture of practices, perceptions, and reference to key emerging themes was presented, providing a background to build a model for swimming analytics. Barriers to the widespread use of IMUs compared with conventional video-based methods were also discussed.

The authors of this paper have published the design for an initial prototype of an acquisition and transmission setup [2] that supports the advancements in the rest of the paper, namely by expanding its capabilities to configure an end-to-end intelligent swimming analytics framework. To the authors’ knowledge, this is the first system employing real-time communications between the swimmer and the coach application.

## 3. Wearable Sensors and Communication Architecture

In swimming, the water environment imposes many constraints on the transmission of sensor data using wireless communications. In the following paragraphs, details of the devised sensors and the communication architecture are presented.

### 3.1. General Architecture

**Figure 1:**
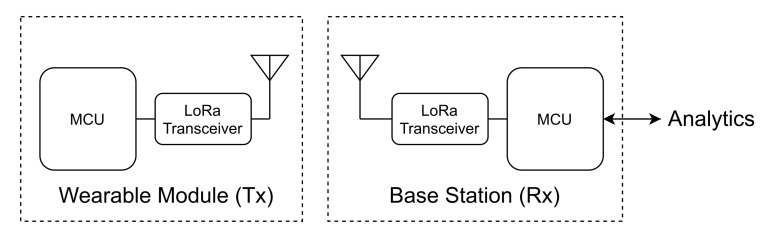
The system architecture: wearable underwater module (**left**) and base station (**right**).

To acquire the relevant data in real time, the system is divided into three modules (see Figure 1):

1. a Wearable Module that acts as the transmitter (Tx);
2. a Base Station that acts as the receiver (Rx); and
3. a Computational Application, that includes the Analytics Engine and the Graphical User interface (GUI).

A 433-MHz half-duplex radio-frequency (RF) communication link, based on the LoRa Peer-to-Peer (P2P) technology (https://lora-alliance.org, access on 29 July 2021), is implemented between the Tx and the Rx. The wearable device (Tx) main component is an MCU that manages the information of the following sensors:

- three-axis accelerometer,
- gyroscope,
- magnetometer,
- heart rate sensor, and
- pulse oximeter sensor ($SPO_{2}$).

To add redundancy to the system, the wearable device also includes a memory card to locally backup all sensor data. Additionally, a haptic stimulation transducer is also included in this module, allowing for an interaction with the swimmer by means of commands received from the base station (Rx).

The wearable device sends data packets to the Rx receiver module that are then delivered to the swimming analytics framework (which will be further detailed) using an asynchronous serial (USART) protocol. The Rx module can also receive commands from the user or from the analytics framework and can send them through the RF link to the wearable device.

**Figure 2:**
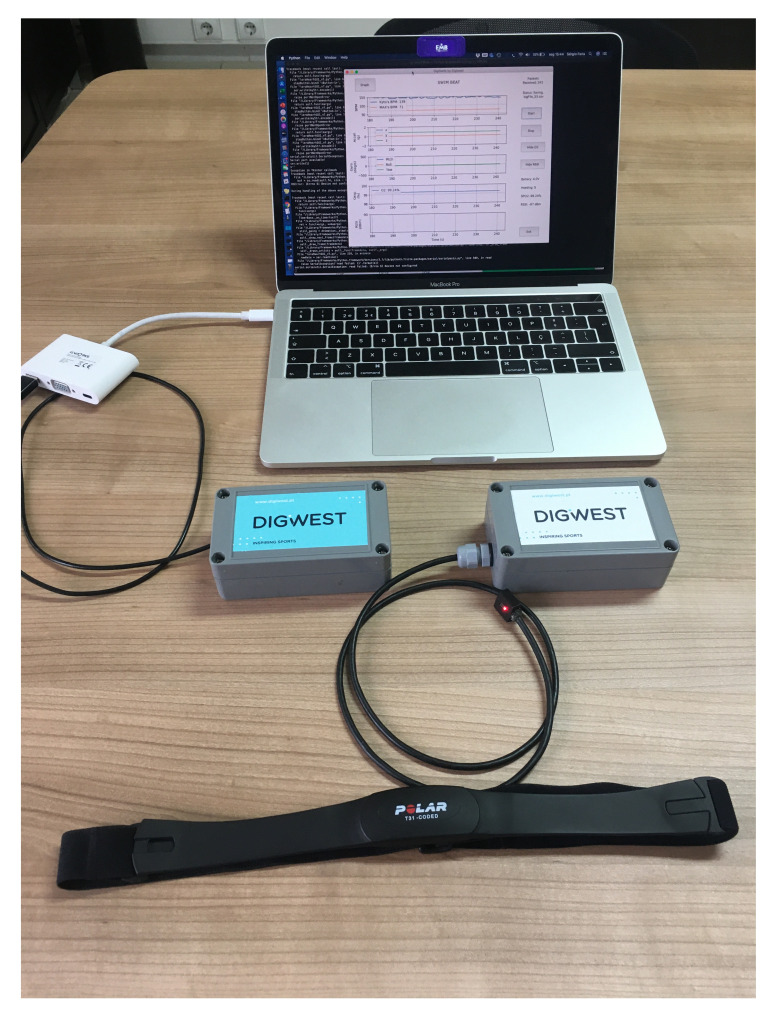
Hardware setup: the wearable device (on the right—white label) and the base station module (on the left—blue label).

The system hardware setup is shown in Figure 2. The base station module (on the left—blue label) connects to the computer running the swimming analytics framework. The wearable device (on the right—white label) has a wired connection to the $SPO_{2}$ sensor and is wirelessly connected to the chest strap that is attached to the swimmer. The heart rate sensor communicates with the Tx module through a 5.3 kHz radio frequency link.

The wearable module uses a watertight enclosure to protect both system modules from water and humidity. This enclosure has the dimensions of 115 mm × 65 mm × 40 mm, weighs 183 g, is 100% waterproof, and allows for a cable to connect the $SPO_{2}$ sensor (outside the case) to the module (inside the case). The MCU used for this system is a board based on an ESP32 microprocessor that interconnects a LoRa transceiver, a Li-Ion battery charger, and an SD card slot, all with a small physical footprint. Two 1300 mAh Li-ion batteries are used to power the system. In order to reduce the case dimensions, the microprocessor board, sensors, LoRa communication system, 5.3 kHz receiver, and batteries are connected using a second, custom-designed, printed circuit board (PCB).

### 3.2. Inertial and Biosignal Sensing

The athlete’s inertial motion data acquired during the training is processed by an Attitude and Heading Reference System (AHRS). An ICM-20948 Motion Tracking chip from TDK InvenSense was used due to the built-in gyroscope, accelerometer, and magnetometer. This system tracks and gathers the swimmer’s body information in a total of nine axes, namely acceleration in the axes X, Y, and Z, heading relative to the geodesic north and angular velocity.

Physiological information such as heart rate, breathing effort, or oxygen saturation is very important to understand if an athlete is training at their physical limits, allowing the coach to adapt and improve the training session parameters, which in turn improves the athlete’s performance. Therefore, in this system, heart rate and oxygen saturation are also measured by means of the two following sensors.

#### **Heart Rate (HR)** 

The wearable module receives the heart beats from a waterproof chest band, in this case, a Polar T31^™^ Coded band, which detects and transmits them through a dedicated 5.3 kHz radio signal. For every detected heart beat, a coded pulse is transmitted by the band. This pulse contains enough information for the 5.3 kHz receiver to distinguish a valid heart beat pulse from a pulse generated by an interfering source. By using the time elapsed between consecutive heart beats, the wearable device calculates the heart rate.

#### **Pulse Oximetry** 

Nowadays, it is possible to measure the hemoglobin concentration in the blood (also known as pulse oximetry or $SPO_{2}$) using noninvasive methods. Unlike the traditional method to measure $SPO_{2}$, using blood analysers and more heavy equipment, pulse oximetry is based on the emission of light towards the blood vessels and measuring the reflection [19]. To this end, it uses a red LED (wavelength of 660 nm) and an infrared LED (wavelength of 940 nm). As blood and hemoglobin concentration affect the amount of reflected light, it can be use to determine the $SPO_{2}$, and heart rate. Such noninvasive sensors are placed on the skin surface, commonly on the fingertips, but can also be installed on the forehead or earlobe. This system uses the MAX30102 sensor from Maxim Integrated to acquire the data, which are then used to calculate $SPO_{2}$, based on the algorithms described in [20,21].

### 3.3. Acquired Data

**Figure 3:**
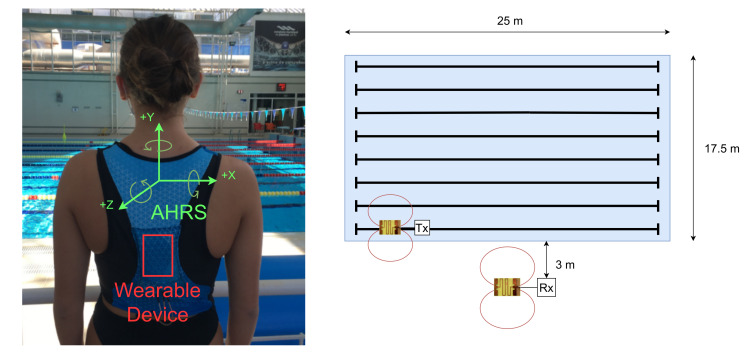
The wearable device placed on the athlete’s back (**left**) and the swimming pool experimental setup diagram (**right**).

The wearable sensors and communication architecture were tested and evaluated at the training swimming pool in the city of Leiria, Portugal. The swimming pool is 25 m long by 17.5 m wide. The tests were performed in lane 1 (farthest distance to the receiver) and lane 8 (closest distance to the receiver). The base station module and the computer running the application were positioned halfway along the pool at an approximate distance of 3 m from lane 8, as can be seen in Figure 3.

The acquisition sampling rate was kept constant at four samples per second, and all data were sent to the Rx module every second. For each athlete, the following data were recorded and stored:

- **Heart rate**: in beats per minute;
- **${\mathrm{SPO}}_{2}$**: in percentage;
- **Acceleration (3 axis)**: between −4 g and 4 g, with a resolution of 0.1 g;
- **Rotation (3 axis)**: between −500 deg/s and 500 deg/s, with a resolution of 1 deg/s; and
- **Heading (3 axis)**: between 0 and 7, representing N, NE, E, SE, S, SW, W, and NW, respectively.

The acquired data was then used to build a stroke classification dataset in order to test and validate the swimming analytics framework, presented in the next section. In the experiments, 10 federated athletes competing at the national level, of both genders, with ages between 15 an 17 years old, were recorded, but data from only four athletes were used in the stroke classification dataset, as not all athletes performed all swimming styles, which are considered the most representative for stroke classification.

The dataset was then manually annotated, and six states were considered: Stopped (0), Butterfly (1), Backstroke (2), Breaststroke (3), Freestyle (4), and Turn (5). All strokes are equally represented in the dataset, as all considered athletes swam the same distances in each swimming style.

## 4. Swimming Analytics Framework

**Figure 4:**
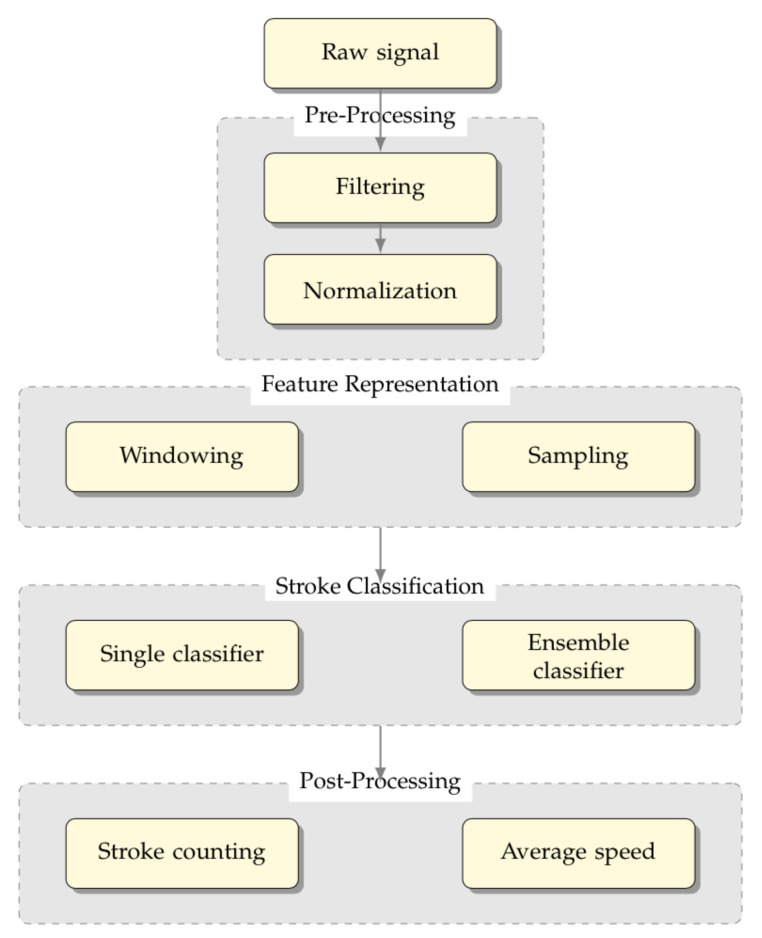
Swimming Analytics Framework that integrates the data acquired by the wearable inertial sensors and biosensors

In this section, we detail the method used for developing the proposed framework, namely a swimming analytics framework is proposed, with the structure depicted in Figure 4.

This system integrates the data acquired by the biosensors and the inertial sensors and allows for online recognition of the swimming style, thus providing important post-analysis insights of the swimmer’s performance for coaching decision support, including average speed and stroke counting.

It is divided into four stages from top to bottom: (i) pre-processing, (ii) feature representation, (iii) stroke classification, and (iv) post-processing.

The pre-processing (i) stage is carried out by acquiring the raw signal provided by the sensors and by adapting their measures into values suitable for being represented computationally. The feature representation (ii) stage allows for the movement representation, given that the single positions acquired by the sensors do not allow for stroke classification. Then, the stroke classification (iii) stage aims to define the best suited learning models to obtain the swimming style classification in real-time.

In the following, each stage of the framework is detailed for a better understanding.

### 4.1. Raw Signal and Pre Processing

In the developed monitoring system, two different kinds of sensors were employed for the heart rate measurement: (i) a heart rate band and (ii) an LED-based SPO2 sensor. The heat rate band is placed around the athletes chest and reads the electromyographic signals originating from the athlete heart. As there are no other sensors or electronics capable of interfering with the heart generated currents, no interference was experienced. The acquired data is then transmitted to the processing unit by means of a 5 kHz radio signal. Such a signal has a very small distance range (of approximately 70–80 cm), and therefore, inter-athlete interference is not likely to occur due to the larger distance between pool lanes. However, even when athletes cross each other on neighboring lanes and, therefore, some radio interference may arise, spurious data is rejected if it falls out of reasonable heart rate values. Such filtering is achieved through the application of a method based in two stages: (i) when heart rate is acquired, outlier heartbeat pulses are ignored, and (ii) a low pass filtering is used afterwards, further reinforcing the ruggedness of the system.

SPO2 levels are acquired by means of an optical LED based sensor, which is inherently immune to electric field interference. Such a sensor is connected via a wire, therefore limiting the interference from the athlete or any other nearby athletes.

However, the data acquired in the wearable sensors and transmitted through the communication system are likely to contain noise. As athletes move in water and especially when they are not swimming, the raw signals acquired by the digital sensors, particularly the heart rate sensor and the magnetometer, need to be pre-processed.

**Figure 5:**
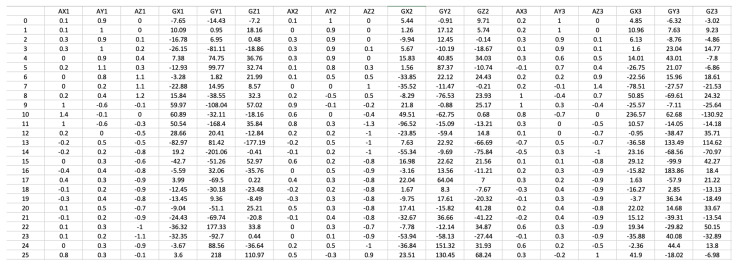
Example of the data acquired by the wearable inertial sensors and biosensors.

Along with the pre-processing filtering applied to the raw signals acquired by the accelerometer and the gyroscope, a mandatory normalization is performed, limiting the output of the accelerometer to the −4 g to 4 g range, and the output of the gyroscope to −500 deg/s to 500 deg/s. Both values are given as double type values. In order to efficiently store the accelerometer data, such data are multiplied by a factor of 10 and saved in 8-bit format. A similar process is applied to the gyroscope output, with the decimal part being discarded and only the integer part saved in 16-bit format. A representation of the acquired data after pre-processing is presented in Figure 5.

### 4.2. Feature Representation

Sensor data acquisition, especially acquired in real time, does not comprise historical data. When representing movement, including swimming, walking, or running, it is not the actual state of each sensor that is relevant but the new position, which is highly dependent on its context, the previous position. Thus, to classify movement, proper feature representation is needed, which cannot rely on single position sensor data as sent by the data acquisition architecture.

The current proposal aims to represent not only the actual swimmer position but also previous positions. Thus, multiple questions arise: How many positions are relevant to store? Are all positions received by the data acquisition architecture relevant for intelligent swimming classification? What is the optimized subset of features that is relevant for swimming stroke classification?

Windowing is the most used and easiest mechanism for feature representation [22,23,24]. It is based on a sliding-window that includes, at each given moment, the most recent and up-to-date data, while the obsolete data points are not taken into account. With this mechanism the up-to-date data are only used for a certain period of time and then are discarded. The choice of the appropriate window size is a critical issue, since we want to keep the relevant previous positions for detection, but we do not want to take into consideration the older positions that carry no information for real-time stroke detection.

Sampling is another relevant mechanism to feature representation [22,25]. It is based on the idea that selecting a subset of unique swimmer positions from the data stream may be enough to represent the overall swimmer position.

### 4.3. Stroke Classification and Post Processing

The swimmer stroke classification problem can be described as a multi-class problem that can be cast as a time-series of swimmer’s positions. It consists on a continuous sequence of instances, in this case, the swimmer’s positions, represented as

(1) $$P=\{p_{1},\cdots ,p_{t}\},$$

where $p_{1}$ is the first occurring instance and $p_{t}$ the latest.

Each instance occurs at a time, usually in equally spaced time intervals, as it is obtained by the data acquisition framework, being characterized by a set of features:

(2) $$D=\{d_{1},d_{2},\cdots ,d_{\left|D\right|}\}.$$

Consequently, the instance $P_{i}$ is represented by the feature vector $\left\{p_{i}1,p_{i}2,\cdots ,p_{i|P|}\right\}$. If $p_{i}$ is a labelled instance, it can be represented by the pair $\left(p_{i},y_{i}\right)$, where $y_{i}\in Y=\left\{y_{1},y_{2},\cdots ,y_{\left|Y\right|}\right\}$ is the class label for instance $p_{i}$.

Notwithstanding being a multi-class problem in its essence, it can be decomposed in multiple binary tasks in a one-against-all binary classification strategy. In this case, a classifier $e^{t}$ is composed by |Y| binary classifiers.

There are different classifiers that can be used for stroke classification. However, when dealing with a problem that requires real-time classification and is highly dependent on the hardware constraints, the framework is two-fold. On one hand, a single classifier strategy is proposed, aiming to perceive which are the best learning models for stroke classification, and on the other hand, a multi-classifier strategy is also proposed. The main idea is that, if computational constraints are not put into place, an ensemble based learning model, using the best performing models from the previous strategy, would present a better performance for stroke classification.

The ensemble model is based on the idea that the use of a committee of classifiers can provide better results than the best of the single classifiers if correctly combined [26,27]. An ensemble is then characterized by

1. a choice of k classifiers and
2. a choice of a combination function, sometimes denominated a voting algorithm.

**Figure 6:**
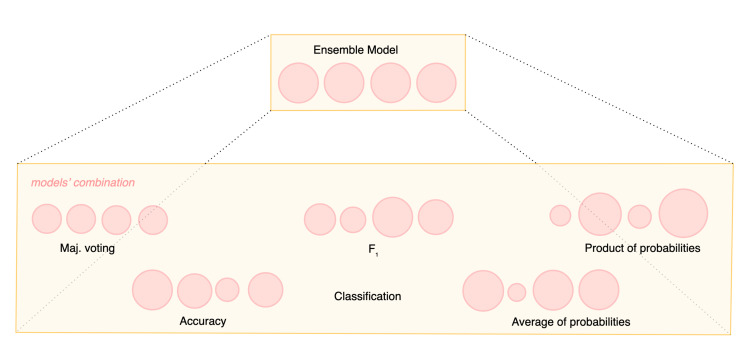
Example of using different metrics when combining models in an ensemble.

The classifiers should be as independent as possible to guarantee a large number of inductions on the data. By using different classifiers, various patterns of errors can be exploited, making the ensemble the best of its baseline models or just the sum (or average) of the parts. The simplest combination function is just a majority voting mechanism with an odd number of baseline classifiers. However, more advanced strategies have been pursued by defining different metrics that can better combine classifiers based on their performance. Figure 6 depicts how different metrics can differentiate the weight of each single classifier in the ensemble final decision. A more complex approach regarding the computational burden is performed by stacking. Stacking is an ensemble-based technique that combines multiple classifiers by using a meta-classifier. The baseline classifiers are trained based on a complete training set, and then, the meta-model is trained on the outputs of the baseline models.

The results of the acquisition and learning processes can be used after post-processing to support coaches’ decisions on athletes’ training and performance. Online recognition of the swimming style and turns can be provided, along with athlete’s performance for coaching decision support. Average speed and stroke counting are considered a relevant insight that can then be provided in real time.

## 5. Experimental Setup

This section starts by detailing the dataset that is tested and evaluated in the proposed approach. Then, the used learning models as well as the performance metrics used to evaluate the proposed approach are characterized. Finally, the post-processing decision tool is detailed.

### 5.1. Learning Models

Multiple classifiers with different characteristics have been tested in order to grasp the best suitable for the problem at hand, namely Naïve Bayes, K-Nearest Neighbors, Decision Trees, and Support Vector Machines.

Besides the setup of the single classifier model, the combination of models in the ensemble model was also handled. The prediction function can use different combining strategies for the output of each classifier in the model combining phase, such as a majority voting strategy, where each classifier contributes evenly to the ensemble model decision. However, other combining schemes can take into account the single model performance to differentiate the weight of each single model to the final decision.

Considering voting, three different combination techniques have been used: majority voting, average of probabilities, and product of probabilities [28]. Regarding stacking, different single classifiers as the meta-classifier were used. The single classifiers used as the meta-classifiers are based on their performance.

Random Forests were also used as it is one of the most well known ensemble-based classifiers, using a collection of single decision trees and merging them together to achieve a more accurate and stable prediction.

### 5.2. Evaluation Metrics

To evaluate the performance of a classifier, evaluation strategies must be defined. One of the most common evaluation strategies is to calculate the accuracy of a classifier, i.e., what is the portion of instances that are correctly assigned to their class. The simplest way of doing so is to split the labelled portion of the dataset into training data and test data. Training data is used in model construction, while test data is used to evaluate the performance of the model. The predictions provided by the model in the test data are then compared with the correct labels, and thus, the accuracy of the classifier is calculated.

Another approach that tends to compensate the rigidity of splits is *cross validation*, where experiments are often repeated with different random splits into training and testing datasets. Usually, the dataset is randomly split and evaluated from 5 to 30 times, and the mean and variance of the criteria are reported. Techniques such as cross validation and *k*-fold validation help guard against randomness, in particular data splits, and allow for sounder results. The *k*-fold validation involves splitting the data in *k* parts (folds), using (k−1) parts for training and the remaining part for testing. This is repeated *k* times, considering all possible testing sets, one at a time. There are multiple advantages, such as considering that every example from the original dataset has the same chance of appearing in the training and testing set, or the ability to perform validations when data are scarce.

In a multi-class problem, as in the stroke classification problem, this can be simplified into a binary decision problem, as each instance can be classified as being in a given class or not. This binary decision setting can be applied by using a one-against-all approach.

In order to evaluate the binary decision task, a contingency matrix can be defined to represent the possible outcomes of the classification, as shown in Table 1.

Traditional measures can be defined based on this contingency table, such as error rate ($\frac{b+c}{a+b+c+d}$) and accuracy ($\frac{a+d}{a+b+c+d}$). However, for unbalanced problems, i.e., problems where the number of positive examples are rather different among classes, or in the case of a binary problem, the number of positive examples is rather different from the negative examples, as often happens in text classification, more specific measures should be defined to capture the performance of each model. Such measures not only usually take into consideration the error but also distinguish between positive and negative errors. Typical examples include recall ($R=\frac{a}{a+c}$) and precision ($P=\frac{a}{a+b}$). Although they can be seen as complementary, recall emphasizes the false negatives while precision is sensitive to false positives. Additionally, combined measures that give a more holistic view of performance in just one value yet considering false negatives and false positives as separate problems are very helpful. Paradigmatic to such measures is the van Rijsbergen $F_{\beta }$ measure [29] that combines recall and precision in a single score:

(3) $$F_{\beta }=\frac{(\beta^{2}+1)P\times R}{\beta^{2}P+R}.$$

$F_{\beta }$ can comprise values from $F_{0}$ to $F_{\infty }$. $F_{0}$ is the same as precision, while $F_{\infty }$ is the same as recall. The most common value assigned to β is β=1, i.e., $F_{1}$, an harmonic average between precision and recall (4).

(4) $$F_{1}=\frac{2\times P\times R}{P+R}.$$

Considering the aforementioned one-against-all strategy and the k-fold validation, a classifier can be trained for each batch that is composed by |Y| binary classifiers, with *Y* being the collection of possible labels. To perceive the performance of the classification for each class, all of the binary classifiers created in all batches must be considered. To evaluate the performance, it is possible to average the results. Two conventional methods are widely used, especially in multi-label scenarios, namely macro-averaging and micro-averaging. Macro-averaged performance scores are obtained by computing the scores for each learning model in each batch and then by averaging these scores to obtain the global means. Differently, micro-averaged performance scores are computed by summing all of the previously introduced contingency matrix values (*a*, *b*, *c*, and *d*) and then use the sum of these values to compute a single micro-averaged performance score that represents the global score. Micro-averaging is particularly relevant in unbalanced problems, while when macro-averaging, all classes are treated as equally contributing to the overall performance of the classifier.

### 5.3. Post-Processing Decision Support Tool

The results of the acquisition and learning processes can be used after post-processing to support coaches’ decisions on athletes’ training and performance evaluation, namely by taking advantage of information as the swimming style, turns, average speed, and stroke counting, which are available in real time.

**Figure 7:**
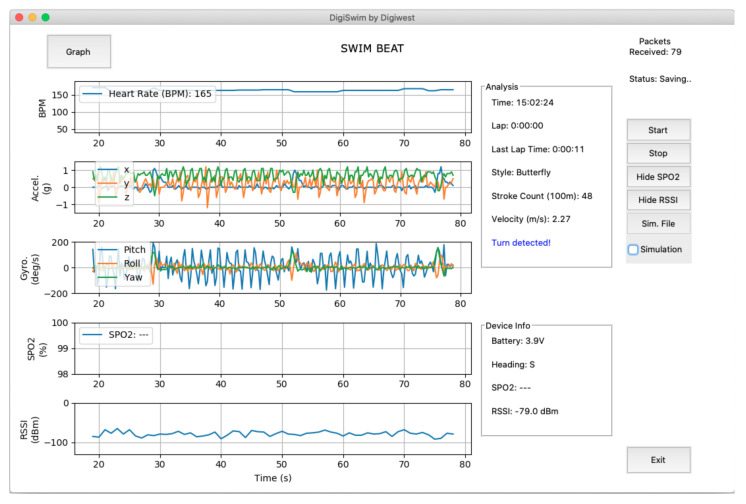
Coaches’ decision support tool with online swimming style and turns recognition.

The framework was deployed through a computational application developed in Python 3.8.0 using Visual Studio Code, allowing for visualisation of the data transmitted by the wearable module in real time as well as analysis, on the right of Figure 7.

**Figure 8:**
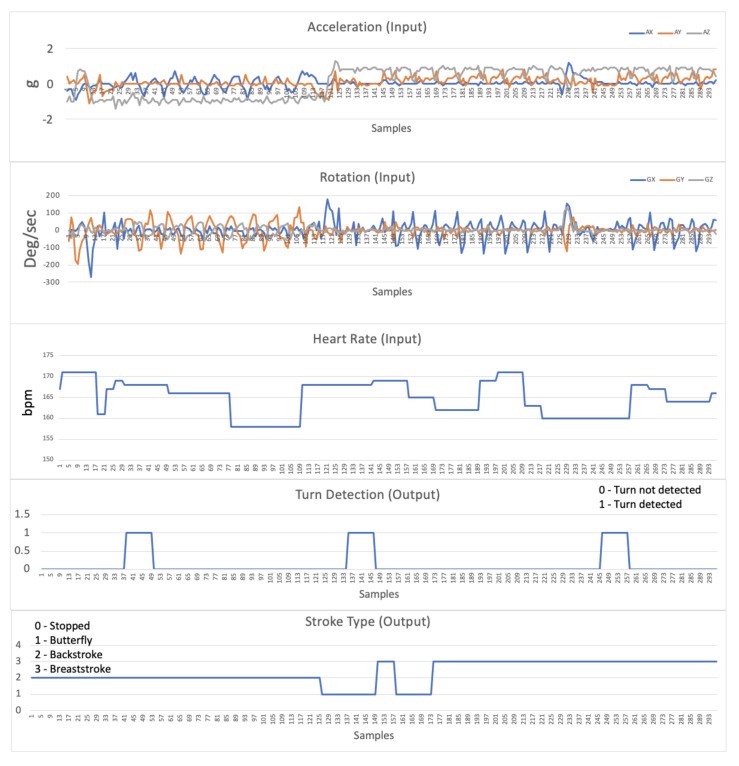
Detail of the data received in real time from the swimmer’s equipment.

Figure 8 presents an example of the real-time received data used by the algorithm. Namely, using accelerometer and gyroscope information, along with heart rate data, it is possible to show turn detection and stroke type, allowing for decision support and correlation between values.

## 6. Experimental Results

In this section, the performance of the approach described in Section 4. Stroke is analyzed on the classification dataset. A 10-fold validation strategy was used along with 10 runs for each learning model.

Table 2 summarizes the performance results obtained by classifying the dataset, considering the $F_{1}$ measure and using different single classifiers, namely Naïve Bayes, K-Nearest Neighbors with different K values (3, 5, and 7), SVM, and two Decision Tree implementations (J48 and Random Tree). Different feature representations were also considered. Bearing in mind that four samples are taken per second, these are tested using a window size from 0.5 s to 4 s (0.5 s, 1 s, 2 s, 3 s, and 4 s, respectively). Additionally, an undersampling approach was tested by considering only the first sample of each second and, again, by using a window size from 1 s to 4 s (1 s und, 2 s und, 3 s und, and 4 s und, respectively).

Considering the results obtained, it is revealed that. for stroke classification purposes, a window size of one second seems to better grasp the swimmer’s movement, as it achieves a 92.42% $F_{1}$ when all other classifiers range from 89.78% to 91.38% .

When comparing only K-Nearest Neighbors classifiers, k equal to five seems to have better classification performance, as it outperforms KNN (k = 3) and KNN (k = 7) in all setups excluding 0.5 s, 2 s und, and 4 s und. Considering only decisions trees, J48 has better classification performance than Random Tree in all setups, except a slight 0.08% decrease in 4 s und.

Table 3 presents the performance results obtained by classifying the dataset considering the $F_{1}$ measure and using different ensemble classifiers. The single classifiers used to compose the ensemble are the same and were chosen based on the classification performance obtained in Table 2. In order to maintain ensemble diversity, the ensemble is composed by Naïve Bayes, KNN (k = 5), as it is the best performer classifier among KNN setups, SVM, and J48, as it is also the best performer classifier among the Decision Tree classifiers. Two combining methods were used: voting and stacking. Regarding voting as combining strategy, three combination rules were tested: majority voting, average of probabilities, and product of probabilities. Concerning stacking, the same four classifiers that compose the ensemble were used to take on the additional role of being the meta-algorithm that is used to grasp each single classifier weight in the final decision of the ensemble. Finally, Random Forest [30] was also tested, as the Random Forest algorithm, is, in its essence, an ensemble classifier of decision trees.

The results outperform all of the results obtained with the use of single classifiers in all feature representation schemes. The performance increases, considering macro-average $F_{1}$, by 2% on average. Comparing only the best performing window size, 1 s, the macro-average $F_{1}$ regarding the output of all single classifiers versus the ensemble ones increases from 92.42% to 94.32%.

Regarding the comparison between the proposed ensemble models, the use of stacking with Naïve Bayes as the meta-classifier has the best performance in four out of the seven feature representation window sizes, namely 0.5 s, 2 s und, 3 s und, and 4 s und.

## 7. Discussion

In this section, the results obtained are discussed and analysed.

Considering the results presented in Table 2, a window size of one second achieves a better classification score regarding the $F_{1}$ measure, which denotes, as already stated, that it seems to better grasp the swimmer’s movement. This might be explained with swimming characteristics and its times. Two seconds and above might be a too long window size, as an athlete can change the stroke type faster than such an interval. On the contrary and considering that half a second only contains two athlete positions, the feature representation might not be informative enough for stroke classification purposes.

Regarding the comparison between the proposed ensemble models, presented in Table 3, the use of stacking with Naïve Bayes as a meta-classifier has the best performance. It is also important to note that those feature representations use less sensor data than others, which seems to denote that stacking with Naïve Bayes, as a meta-classifier, grasps movement better when less features are used, which is an important insight. Considering only the 1 s feature representation, the one that presents the best results regarding stroke classification, Random Forest seems to be the best classifier when using ensembles, which might be related to the robustness of the Random Forest algorithm itself.

In all of the feature representation schemes, the ensemble models outperform the single classifier models. These results attest to the use of ensembles: that using multiple experts, if correctly combined, can lead to better classification performance.

## 8. Conclusions and Future Work

In this paper, an intelligent data analytics system for swimmer performance is presented. The system is supported by wearable inertial sensors and biosensors, which are attached to the swimmer’s body and communicate to the base station through a radio-frequency system.

The system was tested and its performance was evaluated in a real-world scenario at the training swimming pool in the city of Leiria, Portugal.

The results revealed the usefulness of the proposed strategy, as a classification performance of 95.02% was achieved, considering the macro-averaged $F_{1}$ and the Random Forest classifier. It is also relevant to conclude that, when it is possible to support the computational burden of having multiple single classifiers, combined in a ensemble-based classifier, an improved stroke classification could be achieved.

The proposed intelligent swimming analytics system is also designed to provide automatic feedback to the swimmer through an haptic stimulation transducer conveniently located in the wearable device.

Future work will include feature selection with the purpose of determining the features more relevant to stroke classification. Considering the problem at hand, the usage of wearable sensors powered by batteries, and the LoRa-based communication architecture, it is convenient to reduce the bandwidth used in the transmission by sending smaller amounts of data. Consequently, optimizing the amount of transmitted data required to be sent in real-time is of utmost importance.

Additionally, further work may address model interpretability to support the coaching decision process, which can provide a rationale behind model decisions, namely by emphasizing relations between sensor values, e.g., heart rate and $SP0_{2}$, and both the type of stroke/turn and the performance obtained or predicted.

## Figures and Tables

**Table 1:** Contingency table for binary classification problems.

	Class Positive	Class Negative
Assigned Positive	a	b
	(True Positives)	(False Positives)
Assigned Negative	c	d
	(False Negatives)	(True Negatives)

**Table 2:** Performance results of single classifiers using the $F_{1}$ measure for window sizes from 0.5 s to 4 s, with and without undersampling.

	0.5 s	1 s	1 s und	2 s	2 s und	3 s	3 s und	4 s	4 s und
Naïve Bayes	91.82%	93.12%	90.11%	92.43%	92.00%	93.51%	93.32%	91.29%	**92.32%**
KNN (k = 3)	91.89%	92.64%	90.66%	89.68%	89.81%	89.18%	87.72%	90.96%	90.92%
KNN (k = 5)	92.85%	92.91%	90.76%	90.56%	88.48%	90.72%	88.97%	92.10%	90.74%
KNN (k = 7)	**93.19%**	92.38%	90.34%	89.45%	88.40%	90.78%	87.70%	91.78%	90.93%
SVM	91.13%	**93.49%**	88.00%	**92.97%**	**92.23%**	**93.83%**	**93.69%**	**92.91%**	92.31%
J48	90.15%	92.97%	**91.65%**	89.97%	89.84%	88.59%	90.71%	89.39%	87.29%
RandomTree	88.62%	89.41%	88.68%	87.71%	87.71%	87.54%	88.41%	86.55%	87.38%
**Macro-average $F_{1}$**	91.38%	**92.42%**	90.03%	90.40%	89.78%	90.59%	90.07%	90.71%	90.27%

**Table 3:** Performance results of ensemble classifiers using the $F_{1}$ measure for window sizes from 0.5 s to 4 s, with and without undersampling.

	0.5 s	1 s	1 s und	2 s	2 s und	3 s	3 s und	4 s	4 s und
Vote (majority voting)	93.44%	94.86%	92.29%	92.63%	92.22%	93.92%	93.88%	**94.07%**	93.36%
Vote (average of probabilities)	94.13%	94.41%	92.11%	92.81%	91.00%	93.76%	92.89%	93.83%	92.91%
Vote (product of probabilities)	91.86%	93.20%	**92.74%**	92.03%	91.75%	91.21%	92.87%	89.94%	90.20%
Random Forest	93.90%	**95.02%**	92.58%	**94.20%**	92.82%	94.36%	94.09%	93.83%	93.51%
Stacking (Naive Bayes)	**94.32%**	95.00%	91.87%	93.30%	**93.14%**	94.26%	**94.66%**	93.87%	**94.00%**
Stacking (SVM)	94.18%	94.56%	92.56%	92.28%	92.60%	**94.89%**	94.08%	93.35%	93.40%
Stacking (KNN k = 5)	92.86%	93.96%	91.91%	91.69%	91.37%	93.60%	93.88%	92.92%	91.95%
Stacking (J48)	92.02%	93.54%	91.11%	91.65%	89.73%	92.72%	92.32%	91.05%	91.09%
**Macro-average $F_{1}$**	93.34%	94.32%	92.15%	92.57%	91.83%	93.59%	93.58%	92.86%	92.55%

## Data Availability

Not applicable.
